# Proposed practical protocol for flow cytometry analysis of microglia from the healthy adult mouse brain: Systematic review and isolation methods’ evaluation

**DOI:** 10.3389/fncel.2022.1017976

**Published:** 2022-10-19

**Authors:** Sanja Srakočić, Paula Josić, Sebastijan Trifunović, Srećko Gajović, Danka Grčević, Anton Glasnović

**Affiliations:** Croatian Institute for Brain Research, School of Medicine, University of Zagreb, Zagreb, Croatia

**Keywords:** microglia, flow cytometry, digestion methods, cell isolation, brain, microglial cells, demyelination

## Abstract

The aim of our study was to systematically analyze the literature for published flow cytometry protocols for microglia isolation and compare their effectiveness in terms of microglial yield, including our own protocol using sucrose for myelin removal and accutase for enzymatic digestion. For systematic review, the PubMed was searched for the terms “flow cytometry,” “microglia,” “brain,” and “mice.” Three different myelin removal methods (Percoll, sucrose, and no removal) and five protocols for enzymatic digestion (accutase, dispase II, papain, trypsin, and no enzymatic digestion) were tested for the effectiveness of microglia (CD11b^+^CD45^int^ cell population) isolation from the adult mouse brain using flow cytometry. Qualitative analysis of the 32 selected studies identified three most commonly used myelin removal protocols: Percoll, the use of myelin removal kit, and no removal. Nine enzymatic digestion protocols were identified, from which we selected dispase II, papain, trypsin, and no enzymatic digestion. A comparison of these myelin removal methods and digestion protocols showed the Percoll method to be preferable in removal of non-immune cells, and superior to the use of sucrose which was less effective in removal of non-immune cells, but resulted in a comparable microglial yield to Percoll myelin removal. Digestion with accutase resulted in one of the highest microglial yields, all while having the lowest variance among tested protocols. The proposed protocol for microglia isolation uses Percoll for myelin removal and accutase for enzymatic digestion. All tested protocols had different features, and the choice between them can depend on the individual focus of the research.

## Introduction

Microglia are resident macrophages in the central nervous system (CNS), where they play an important role in inflammation and reparatory processes (Li and Barres, 2018). They have their origin in a unique stem cell in the yolk sac, whose cell descendants during fetal development migrate to the CNS. This sets them apart from the ectoderm-derived cells of the nervous system. Extensive research has aimed to clarify the role of microglia in different CNS pathologies, including brain ischemia and neurodegenerative diseases (Fakhoury, 2018; Jiang et al., 2020; Liu et al., 2020).

Flow cytometry is an indispensable method for the quantification and identification of the cells in any tissue sample, including brain samples containing microglia (Bohlen et al., 2019; Brioschi et al., 2020; Calvo et al., 2020). When being prepared for flow cytometric analysis, already mechanically processed tissue is frequently digested with enzymes as chemical agents. As the standard methods of single-cell suspension preparation, these treatments disrupt the intercellular junctions and the extracellular matrix. In tissue processed in this way, the cytometer can detect the signals of each individual cell passing through a laser beam (McKinnon, 2018). A specificity of brain samples for microglia analysis is that they contain an abundant amount of myelin. Myelin can be removed so that it does not interfere with flow cytometric analysis.

In flow cytometry, cells are detected after incubation with fluorescently labeled antibodies against markers expressed on their membrane. To identify microglia, a combination of anti-CD 11b (CD–cluster of differentiation), and anti-CD45 antibodies is usually used. Fluorophores adhere to the cells and start to emit light upon activation with laser. The light is detected by a detector, allowing us to gain insight into the number and phenotype of the cells (McKinnon, 2018; Molina Estevez et al., 2019; Agalave et al., 2020).

As the standard methods of sample preparation for flow cytometry are not adequate for brain samples, they need to be complemented by myelin removal methods to achieve a sufficient separation rate (Skog et al., 2019; Chometon et al., 2020). They may also be too aggressive, leading to a loss of surface antigens and inadequate results (Skog et al., 2019; Chometon et al., 2020). Therefore, we aimed to compare different methods of CNS tissue digestion and myelin removal to find an optimal protocol that would adequately affect brain samples and yield reliable and reproducible results. The methods to be verified in the laboratory setting were selected after a systematic literature search for the most commonly used protocols. Along with protocols identified through the literature search, we also tested our own protocol where sucrose was used for myelin removal and accutase for tissue digestion in order to identify the optimal practical protocol for microglia isolation and flow cytometry analysis from the adult mouse brain.

The quantitative analysis of the published studies identified by the systematic literature search indicated three procedures of myelin removal: Percoll (a silica-based solution), myelin removal kits, and no-removal protocol. In the experimental part of our study, we compared the Percoll protocol, sucrose protocol, and no-removal protocol. In addition, nine different enzyme digestion protocols were identified in the literature search. We experimentally tested four enzyme solutions for tissue digestion: accutase, papain, dispase II, and trypsin, and compared them with the protocol without enzymatic digestion. The choice of myelin removal method affected the overall microglial yield far more than the choice of digestion enzyme. The Percoll based myelin removal and accutase digestion proved to be the best protocols. However, all of the tested protocols had different advantages and disadvantages, and the choice between them should depend on the individual focus of the research.

## Materials and methods

### Systematic review of the literature

#### Search strategy

The systematic review was conducted in accordance with the PRISMA statement (Liberati et al., 2009) and SYRCLE guidelines (de Vries et al., 2015). We searched for the studies published in the last 5 years (starting with 2017) that employed flow cytometry for the analysis of microglial cells isolated from the adult mouse brain. The PubMed was systematically searched on May 24, 2022. Titles, abstracts, and keywords were searched using the following terms:

(1) “Flow cytometry”
(2) “Microglia”
(3) “Brain” OR “CNS”
(4) “Mouse” OR “mice” OR “murine”

All search terms were applied together, linked with the Boolean operator “AND.” The Medline code of search was as follows: (flow cytometry) AND (microglia) AND [(brain) OR (CNS)] AND (mouse OR mice OR murine). No language barriers were applied. The search was conducted by two researchers independently.

#### Study selection

After identification of the publications, the following inclusion criteria were applied for the initial screening of the abstracts and full-text screening of the identified studies:

(1) the study reported new, original results (primary research article),
(2) flow cytometry was employed for the analysis of microglial cells,
(3) adult mice of any sex were used as a model,
(4) microglia were isolated from the brain tissue.

The studies that did not use a mouse model and the studies performed on perinatal or neonatal mice were excluded. Furthermore, studies were excluded if microglia were isolated only from cell culture (not from isolated brains) or from nervous system tissue different from the brain (e.g., spinal cord). Screening was performed by two researchers independently, and all discrepancies were resolved through discussion with a third researcher.

#### Data extraction for qualitative synthesis

The content of the selected publications was analyzed in detail. We extracted information on: (1) animal models used (animal strain, age, and sex); (2) the protocol for microglia isolation (myelin removal method, type of enzymatic digestion, duration of enzymatic digestion, and concentration of digestion enzymes); (3) flow cytometry (identification of microglial population, detailed gating strategy, use of viability dye, and additional markers). Data were extracted by two researchers, and all differences were resolved through discussion with a third researcher.

### Experimental study

#### Animals

The experiments were performed on adult (12–16 weeks old, weight 25 ± 3 g) male albino inbred mice (strain C57BL/6-Tyr*^c–Brd^*/J) from the animal facility of the School of Medicine, University of Zagreb. Animals were housed under standard conditions and 12-h light/dark cycle, with water and food *ad libitum*. All procedures were approved by the Ethics Committee of the School of Medicine, University of Zagreb, and by the Ministry of Agriculture of the Republic of Croatia. The procedures conformed to the Ethical Codex of the Croatian Society for Laboratory Animal Science and to the EU Directive 2010/63/EU on the protection of animals used for scientific purposes.

The mice were randomly divided into seven experimental groups with 8 animals in each group (*N* = 56). In three experimental groups, different myelin removal methods were tested: Percoll, sucrose and no removal; in all of these groups tissue digestion was performed using accutase. In four experimental groups, different digestion protocols were tested (dispase II, trypsin, papain, and no enzymatic digestion) with the Percoll protocol as a myelin removal method of choice. All brains were processed individually.

Finally, four mice were used for additional phenotyping with immune cell markers (MHC-II, Ly6C, and Ly6G), as well as for the additional testing of blocking protocol (Fc block versus FBS) and labeling-incubation temperature. In this experiment, tissue was digested with accutase, myelin was removed using Percoll and all four brains were pooled.

The total number of animals used in this study was 60.

#### Microglia isolation

Mice were anesthetized and transcardially perfused with cold phosphate buffered saline (PBS, pH 7.4). The brains were isolated and placed in 2 mL ice-cold Hank’s Balanced Salt Solution (HBSS, Capricorn Scientific, Ebsdorf, Hessen, Germany), where they were mechanically cut into parts as small as possible with a surgical scalpel. The brains were digested using different digestion enzymes for each experimental group: accutase (StemPro Accutase™, Gibco, Thermo Fisher Scientific, Waltham, Massachusetts, MA, USA), papain (1 mg/mL, Sigma Aldrich, St. Louis, Missouri, MO, USA), dispase II (2.4 mg/mL, Sigma Aldrich, St. Louis, Missouri, MO, USA) and trypsin (0.5%, Sigma Aldrich, St. Louis, Missouri, MO, USA). To each brain, 2 mL of particular digestion enzyme was added. All samples were incubated for 30 min at 37°C. After incubation, the enzymes were inactivated by adding 4 mL of Dulbecco’s Modified Eagle Medium (DMEM, Gibco, Thermo Fisher Scientific, Waltham, Massachusetts, MA, USA). In this step, DMEM was also added to the control protocol without digestion. The samples were spun down, brain tissue was collected and washed with 2 mL of HBSS. Next, tissue was mechanically homogenized using a spatula with the aim of transforming clumps of tissue into a cell suspension. The debris was removed by passing the cell suspension through a 70-μm cell strainer (Falcon™, Gibco, Thermo Fisher Scientific, Waltham, Massachusetts, MA, USA). To ensure the adequate collection of cells, the strainers were additionally washed four times with 1 mL of HBSS. Finally, the cells were spun down.

#### Myelin removal methods

##### Percoll protocol

The first protocol of myelin removal was performed in 15 mL centrifuge tubes with three different solution gradients of Percoll (Sigma Aldrich, St. Louis, Missouri, MO, USA) dissolved in 10X HBSS: 30% (4 mL), 37% (4 mL) and 70% (4 mL) (Calvo et al., 2020). After debris removal, the cells were dissolved in 37% isotonic Percoll solution. Percoll gradient was formed as follows: the bottom layer consisted of 70% gradient solution, the middle layer consisted of 37% gradient solution, and the top layer consisted of 30% gradient solution. These gradients were covered by 2 mL of HBSS. The samples were centrifuged for 40 min at 300 g and 18°C (Universal 320R, Hettich, Germany). The centrifugation was performed on 18°C to ensure that the density of Percoll gradients remained intact. Similarly, to protect the gradients the acceleration was set to minimum while the braking settings on the centrifuge were disabled. After centrifugation, demyelinated cells were located on the border between the 37 and 70% Percoll gradient, while myelin was located at the top of the tube, in HBSS. 3 mL of demyelinated cells were collected from the 37 and 70% gradient interface and diluted two times with 6 mL of HBSS to remove the remaining Percoll.

##### Sucrose protocol

Myelin removal was performed with 0.9 M sucrose (Sigma Aldrich, St. Louis, Missouri, MO, USA) dissolved in HBSS. This particular concentration of sucrose was chosen since it had the same osmolality as isolated cells. The cells were resuspended in 8 mL of 0.9 M sucrose solution and centrifuged for 40 min at 300 g and 18°C. After centrifugation, the supernatant was removed, and 3 mL of pellet containing demyelinated cells was collected. The samples were diluted two times with 6 mL of HBSS.

##### No myelin removal protocol

Myelin removal protocol was not performed. After debris removal by cell strainers, the samples were washed with HBSS and stained with antibodies.

#### Flow cytometry and gating strategy

In the experimental groups assessing the efficiency of myelin removal method or the choice of enzyme for tissue digestion after myelin removal, the cells were labeled using CD45 (rat anti-mouse, conjugated with APC-allophycocyanin, 1/200, LOT: B289585, BioLegend, San Diego, California, CA, USA) and CD11b antibodies (rat anti-mouse, conjugated with Alexa Fluor^®^ 488, 1/200; LOT: B254608, BioLegend, San Diego, California, CA, USA). Antibodies were diluted in 2% fetal bovine serum (FBS, Gibco, Thermo Fisher Scientific, Waltham, Massachusetts, MA, USA) in PBS solution as 1:100. For labeling, samples were incubated for 30 min at room temperature, in 50 μL total volume, in PBS with 2% FBS to block non-specific binding of Fc receptors. After incubation, the samples were washed in 2 mL of 2% FBS solution and transferred into FACS tubes (Falcon™, Gibco, Thermo Fisher Scientific, Waltham, Massachusetts, MA, USA). 7AAD (7-Aminoactinomycin D, BioLegend, San Diego, California, CA, USA) viability dye was added into a final concentration of 0.25 μg/mL.

For the last experimental group, the pooled samples were separated into four sub-groups. In the first and second sub-group, Fc block was applied; in the first sub-group, incubation with antibodies was performed at room temperature, while the second sub-group was incubated on ice. In the third and fourth sub-group, Fc block was not utilized. The third sub-group underwent incubation with antibodies on room temperature, while the fourth group was incubated on ice. In the sub-groups were the Fc block was implemented prior to antibody staining, the cells were incubated for 7 min with the TruStain fcX™ anti-mouse CD16/32 (1 μg per sample, BioLegend, San Diego, California, CA, USA). In this experiment, the cells of all sub-groups were stained with the MHC-II (rat anti-mouse, conjugated with PE, 1/400; BioLegend, San Diego, California, CA, USA), Ly6C (rat anti-mouse, conjugated with PE, 1/300; BioLegend, San Diego, California, CA, USA) and Ly6G (rat anti-mouse, conjugated with PE-Cy7, 1/100; BioLegend, San Diego, California, CA, USA) antibodies, along with the CD45 and CD11b antibodies. The antibodies were dissolved in 2% FBS in PBS. The samples were incubated with the antibodies for 30 min, followed by washing in 2% FBS and addition of 7AAD viability dye (0.25 μg/mL).

**FIGURE 1:**
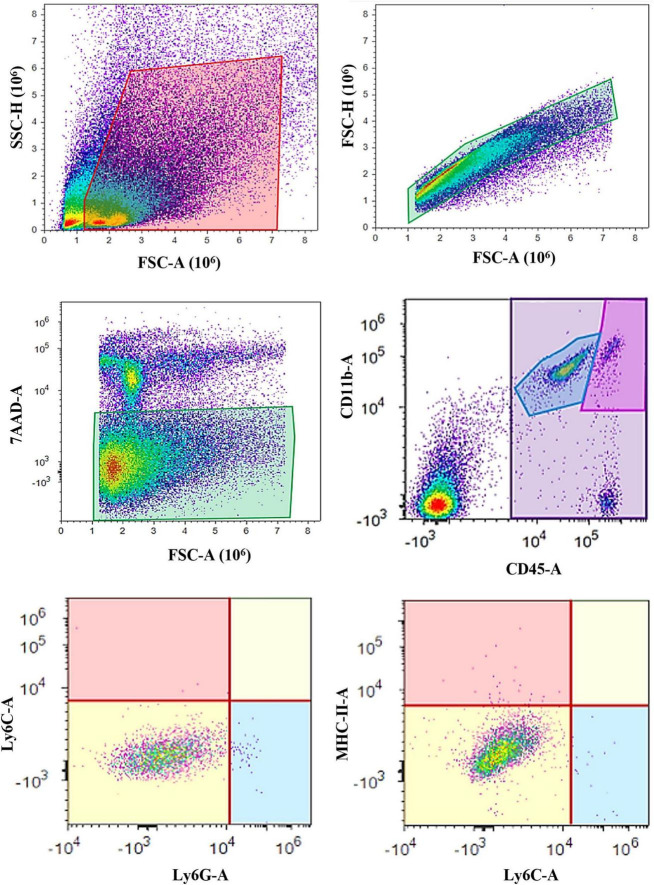
Gating strategy for microglia analysis using flow cytometry. The cells were first sorted based on their forward (FSC) and side (SSC) scatter, followed by the single-cell gate (FSC-A and FSC-H) and live cells separation (7-AAD^–^). Next, the resting microglia were defined as the CD11b^+^CD45^int^ cell population, while the CD11b^+^CD45^high^ cells represented activated microglia and macrophages. Both the CD11b^+^CD45^int^ and CD11b^+^CD45^high^ populations were further assessed for Ly6C and Ly6G surface markers. MHC-II expression was additionally evaluated on the Ly6C^–^Ly6G^–^ cell populations.

Flow cytometry was performed using Attune^®^ Acoustic Focusing Cytometer (Thermo Fischer Scientific, Waltham, Massachusetts, MA, USA) with excitation lasers of 488 nm and 638 nm, and collecting filters for wave lengths of 530 ± 30 nm (for CD11b antibody conjugated with Alexa Fluor^®^ 488), 574 ± 26 (for MHC-II antibody conjugated with PE), 660 ± 20 nm (for CD45 antibody conjugated with APC), 690 ± 50 nm (for 7AAD viability dye), 780 ± 60 (for Ly6G antibody conjugated with PE-Cy7), and 780 ± 60 (for Ly6C antibody conjugated with APC-Cy7). The gating strategy was to separate the cells based on forward (FSC) and side scatter (SSC) in order to remove cell aggregates and small debris, followed by single-cell gate (FSC-A and FSC-H). Next, live cells were defined as 7AAD-negative population since dead cells were excluded, and these cells were further analyzed for CD45 and CD11b expression. Resting microglial population was defined as CD11b^+^CD45^intermediate^, and activated microglia/macrophages were defined as CD11b^+^CD45^high^ population (Figure 1). Finally, CD11b^+^CD45^int^ and CD11b^+^CD45^high^ populations were assessed for Ly6C, Ly6G, and MHC-II expression. First, both cell populations (CD11b^+^CD45^int^ and CD11b^+^CD45^high^) were assessed for Ly6C and Ly6G expression. Then, the Ly6C^–^Ly6G^–^ cell populations were additionally evaluated for MHC-II expression (Figure 1).

#### Statistical analysis

Sample size was calculated using a power test with 80% confidence level. Normality of data distribution was assessed with a Shapiro-Wilk test. Differences between the experimental groups were assessed with one-way ANOVA for non-repeated measures and Dunnett’s test for *post hoc* analysis. Statistical analysis was performed with R software. Differences were considered statistically significant if *P* < 0.05. For experimental groups with similar means, coefficients of variance (CV, defined as the ratio of the standard deviation to the mean) were calculated to determine the extents of variability. All data are presented as mean ± standard deviation.

## Results

### Systematic review of the literature

#### Study selection process

**FIGURE 2:**
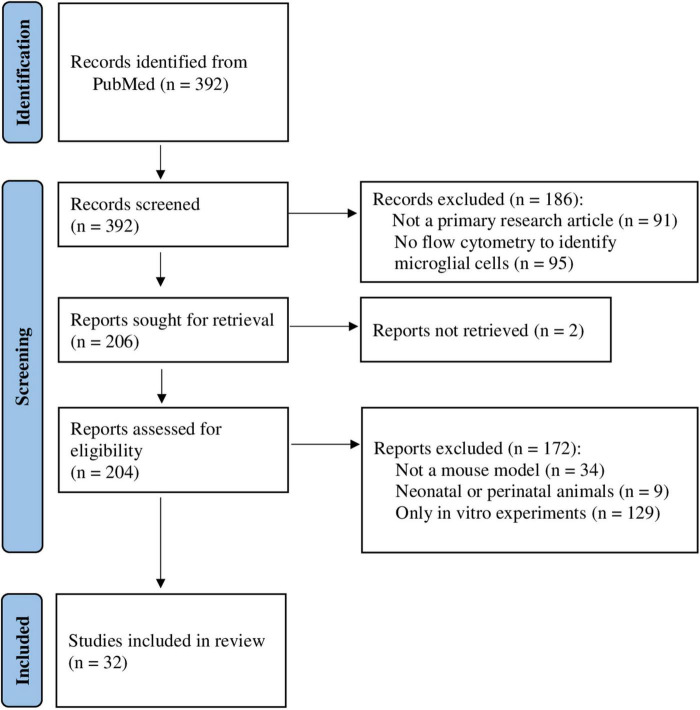
Flow chart of the study selection process for the identification of studies included in the systematic review. Database search yielded 392 results (2017–2022), and after applying inclusion and exclusion criteria, 32 studies were selected for qualitative synthesis.

The literature search identified 392 studies (Figure 2). After initial screening of the titles and abstracts, 186 publications were excluded: 91 were not a primary research article and 95 did not perform flow cytometry to identify microglial cells. Moreover, for two publications full-text versions were not retrieved. A total of 204 publications were assessed for eligibility. Of these, 172 publications were excluded: 34 did not use a mouse model, 9 used perinatal or neonatal animals, and 129 performed *in vitro* experiments only. Finally, 32 publications (Supplementary Table 1) were included in a qualitative synthesis. Since 5-year publication date restriction was applied, the oldest publications were from 2017, and the most recent from 2021. No publications from 2022, when the search was performed, fulfilled the inclusion/exclusion criteria. Most of the selected studies were published in 2020.

#### Characteristics of animals used in flow cytometry experiments

**FIGURE 3:**
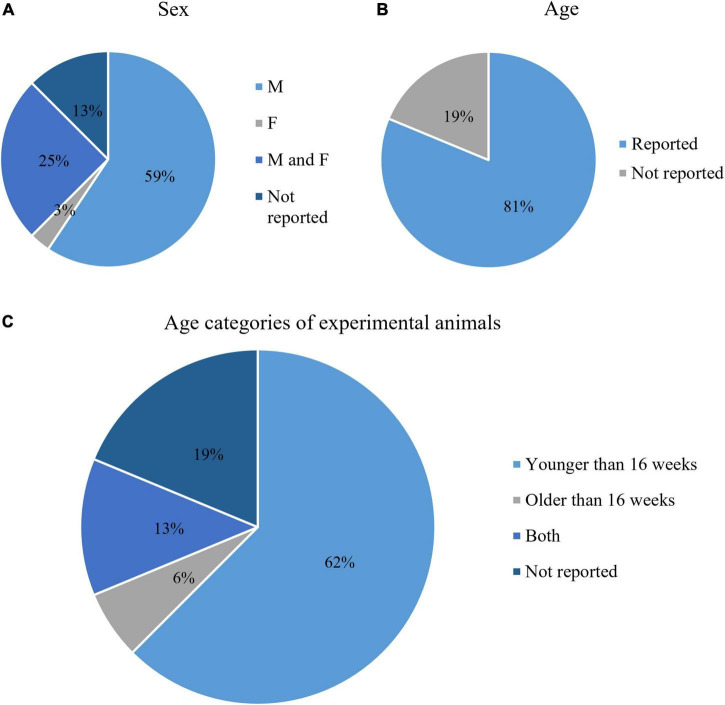
Characteristics of the animals used in the included studies: Animal sex **(A)**, whether or not animal age was reported **(B)**, and age categories **(C)**.

Most of the analyzed studies used mouse strains with C57BL/6 background (91%, 29/32), with various genetic mutations. Four (13%) studies did not report the sex of the experimental animals. The majority of the studies used only male mice (68%, 19/28), one study used only female mice, and 8 (29%) studies used mice of both sexes (Figure 3A). The age of the animals was reported in 26/32 (81%) studies (Figure 3B). For the purposes of this study, we categorized the age as younger than 16 weeks and older than 16 weeks. Most of the selected studies used animals younger than 16 weeks (77%, 20/26), 8% (2/26) used animals older than 16 weeks, while 15% (4/26) combined in their samples both age categories (Figure 3C). To summarize, the most common experimental model for microglia analysis by flow cytometry experiments was male mice younger than 16 weeks with C57BL/6 genetic background.

#### Protocols for microglia isolation and flow cytometry analysis

**FIGURE 4:**
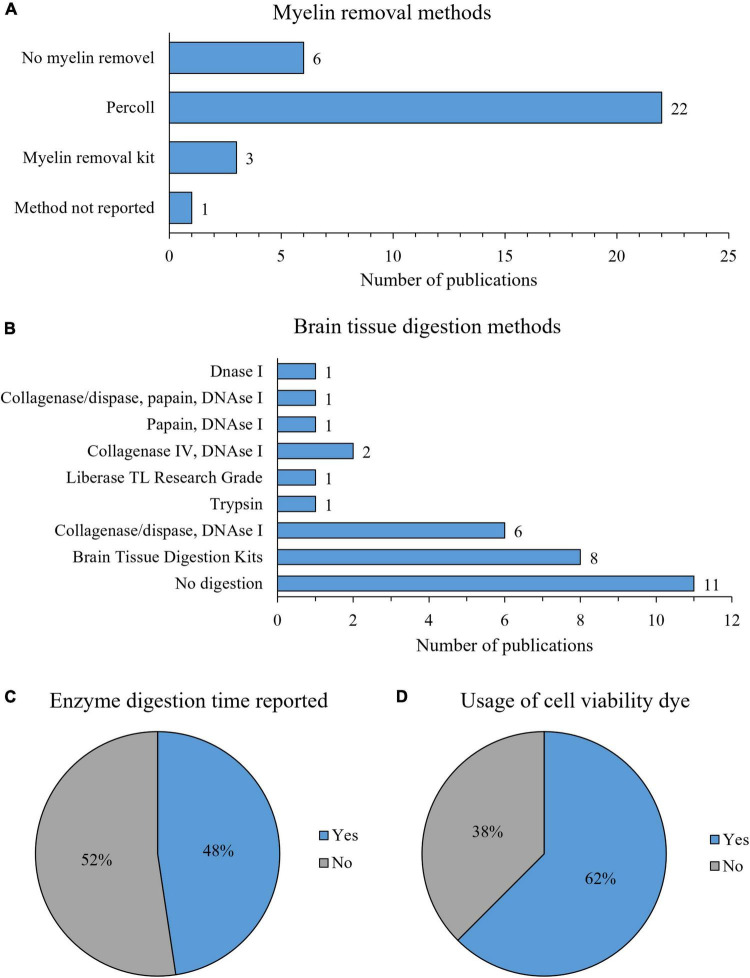
Characteristics of microglia isolation protocol: myelin removal methods **(A)**, brain tissue digestion protocols **(B)**, reporting of enzyme digestion time **(C)**, and usage of viability dye for flow cytometry experiments **(D)**.

In the selected 32 studies, we identified three different myelin removal methods (Figure 4A). Myelin removal with Percoll was most commonly applied (69%, 22/32). However, studies differed on concentrations of Percoll solutions used. Some used a single concentration of Percoll, while others used two or three different Percoll concentrations, creating gradients. Six studies (19%) did not use any myelin removal method, three (9%) used commercially available kits for myelin removal, and in one (3%) the myelin removal method was used but not disclosed (Figure 4A). Three studies using myelin removal kits employed three different commercially available kits.

Various digestion enzymes and their combinations were used for mouse brain tissue digestion, including collagenase, dispase, liberase, trypsin, papain and DNAse I. We identified nine different enzymatic digestion protocols (Figure 4B). Eleven (34%) studies reported omitting enzymatic digestion, while the most commonly used protocols were brain tissue digestion with commercially available kits (25%, 8/32), digestion with a combination of collagenase/dispase and DNAse I (19%, 6/32) and digestion with a combination of Collagenase IV and DNAse I (6%, 2/32) (Figure 4B). Other digestion protocols (DNAse I, trypsin, Liberase, papain in combination with DNAse I and collagenase/dispase with papain and DNAse I) were used in only one study (Figure 4B). As for commercially available kits, two different kits were used: Adult Brain Dissociation Kit (37.5%, 3/8) and Neural Tissue Dissociation Kit (67.5%, 5/8), both by Miltenyi Biotec. All of the studies that used enzymatic digestion reported enzyme concentration, except studies that used commercially available digestion kits. However, only 48% (10/21) of the studies reported the duration of enzymatic digestion (Figure 4C).

All analyzed studies reported using CD11b (marker of myeloid cell line) and CD45 (marker of immune cells) for microglia selection. In all studies, microglial population was defined as a population expressing CD11b and having a medium expression of CD45 (CD11b^+^ CD45^int^). Gating strategies were also similar. All studies first separated the cells using forward (FSC) and side (SSC) scatter. The number of microglial cells was then determined either in the total cell population or in the live-cell population depending on whether cell viability dye was used. Most of the studies (63%, 20/32) reported using cell viability dye (Figure 4D). The most commonly used cell viability dyes were carboxylic acid succinimidyl ester (20%, 4/20 studies), Zombie Aqua (15%, 3/20 studies), and 7AAD (10%, 2/20 studies). Other cell viability dyes were used in only one study (Supplementary Table 1). These protocols can be applied not only for microglia isolation but for the isolation of other CNS cells. In the analyzed studies, additional detection antibodies were used to detect neutrophils and monocytes (Ly6G and Ly6C, respectively), astrocytes (ACSA-2, GFAP), oligodendrocytes (O4) and lymphocytes (CD4, CD8, and CD19), in the same suspension with microglial cells (additional markers used in the experiments are listed in Supplementary Table 1). In addition, the expression of other microglial receptors, such as TMEM119, MHC-II, CD68, was analyzed. Flow cytometry was also implemented to determine the number of various interleukins (TNF-α, IFN-γ, IL-1β, IL-4, IL-10) related to neuroinflammation.

To summarize, the method of choice for myelin removal was Percoll. Most commonly, no enzymatic tissue digestion was performed. Regarding the overall protocol (a combination of myelin removal method and tissue digestion method), the identified studies most commonly used demyelination with Percoll without tissue digestion (25%, 8/32), followed by myelin removal with Percoll with tissue digestion using kits (16%, 5/32) and myelin removal with Percoll with digestion using collagenase/dispase and DNAse I (13%, 4/32). Microglial population was always defined as CD11b^+^ CD45^int^ cells, and cell viability dye was implemented into protocol in majority of the studies.

### Experimental study

#### Impact of different myelin removal methods on microglial yield on flow cytometry

We compared the microglial yield of three myelin removal methods: Percoll, sucrose and no myelin removal. In these experimental groups, enzymatic digestion was performed with accutase. We assessed how different myelin removal protocols affected the frequency of live cells (7AAD^–^ cell population), immune cells (pan-leukocytes, CD45^+^ population), overall resting microglia (CD11b^+^CD45^int^), as well as activated microglia and macrophage (CD11b^+^CD45^high^) yield in total cells isolated from the adult mouse brain. To assess the specificity of different myelin removal methods for immune cell isolation, we compared the number of pan-leukocytes (CD45^+^ cells) expressed as the percentage of isolated live-cell population in total cells count. This cell population from the adult mouse brain includes resting microglial cells, activated microglia and macrophages, and lymphocytes infiltrated into the brain from peripheral blood (found within the CD45^+^CD11b^–^population). The number of isolated microglial cells and cell frequency were assessed. While the absolute number of microglial cells provided information about protocol effectiveness, cell frequency assessment allowed for a comparison of different microglia isolation protocols. CV was calculated as a measure of protocol reliability since reduction of animal use in experiments is encouraged.

**FIGURE 5:**
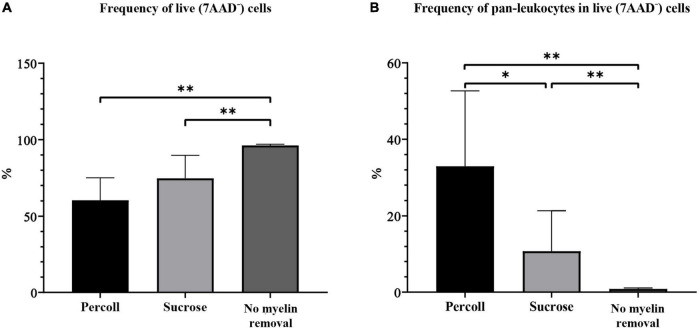
The frequency of live cells **(A)** and pan-leukocytes **(B)** on flow cytometry obtained by different myelin removal methods. *P < 0.05; ^**^P < 0.01. *N* (mice per group) = 8.

The frequency of live cells was significantly higher in the no myelin removal group (96.13% ± 0.31) than in the Percoll (60.40% ± 4.88, *P* < 0.01) and sucrose group (74.67% ± 5.00, *P* < 0.01) (Figure 5A). The percentage did not differ significantly between Percoll and sucrose myelin removal methods (*P* = 0.16).

The frequency of pan-leukocytes was significantly higher in the Percoll group (32.94% ± 6.58) (Figure 5B) than in the sucrose (10.76% ± 3.53, *P* = 0.02) and no-demyelization group (0.86% ± 0.09, *P* < 0.01) (Figure 5B).

**FIGURE 6:**
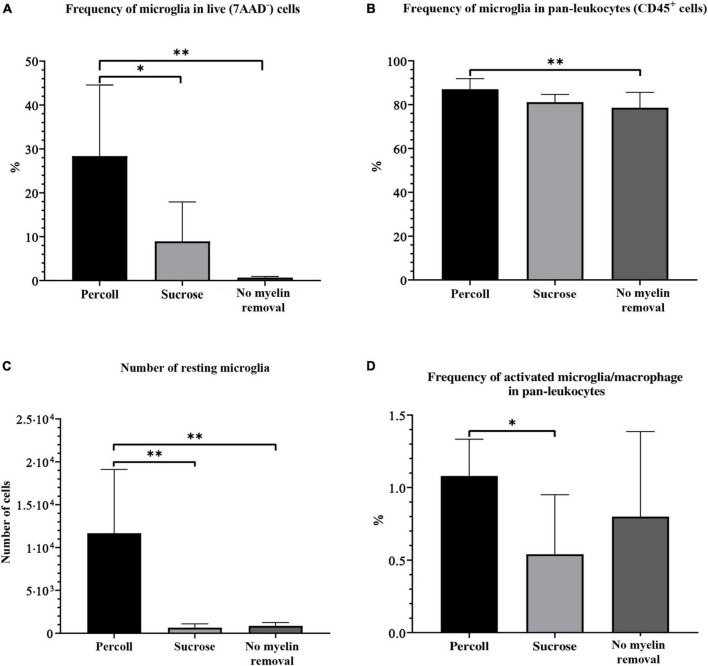
The percentage of resting microglia in the live-cell population **(A)**, pan-leukocyte population **(B)**, absolute number of resting microglia **(C)**, and percentage of activated microglia and macrophages **(D)** on flow cytometry obtained by different myelin removal methods. *P < 0.05; ^**^P < 0.01. *N* (mice per group) = 8.

The frequency of microglia was calculated in the CD45^+^ population in all (live, 7AAD^–^) cells. We also assessed the absolute number of microglial cells. The percentage of resting microglia in live cells (28.37% ± 5.40) and the absolute number (11673 ± 2478) of microglia were significantly higher in the Percoll group than in the sucrose (frequency in live cells: 8.94 ± 8.93%, *P* = 0.02; absolute number: 647.20 ± 450.30, *P* < 0.01) and no myelin removal (frequency in live cells: 0.68 ± 0.23%, *P* < 0.01; absolute number: 856.30 ± 393.70, *P* < 0.01) group (Figures 6A,C). However, the frequency of resting microglia in the CD45^+^ population did not differ between the Percoll and sucrose groups (*P* = 0.06) (Figure 6B). Once again, the Percoll protocol yielded the highest frequency (87.07% ± 1.60), followed by the sucrose (81.15% ± 1.18) and no myelin removal protocols (78.60% ± 2.50). All experimental groups had similarly low CV (5.52% Percoll, 4.37% sucrose, and 8.98% no myelin removal).

The frequency of activated microglia and macrophages was significantly lower in the sucrose group (0.54% ± 0.14) compared to the Percoll group (1.08% ± 0.09). The frequency in the no myelin removal group (0.80% ± 0.21) was lower than in the Percoll group, but the difference was not significant (*P* = 0.42) (Figure 6D). In addition, the Percoll group had a lower CV (23.44%) than the no myelin removal group (73.34%), which makes it a more reliable method for activated microglia and macrophage isolation.

To summarize, the choice of myelin removal method greatly affected cell yield. The Percoll protocol was by far the best method for microglia isolation from the adult mouse brain, with the highest cell yield and a low CV in almost every assessed category. Although the Percoll protocol yielded fewest live cells, the number of pan-leukocytes, including microglia, was highest. The sucrose protocol was less effective in terms of pan-leukocyte frequency, but the number of microglia was comparable to the Percoll protocol. The no myelin removal protocol had the lowest microglial yield, although this method had the advantage of the highest cell viability.

#### The use of different digestion enzymes alters microglial yield

Since the Percoll protocol yielded the best results, it was used in all the experiments testing different types of enzymes: accutase, papain, dispase II, trypsin, and the protocol without enzymatic digestion.

**FIGURE 7:**
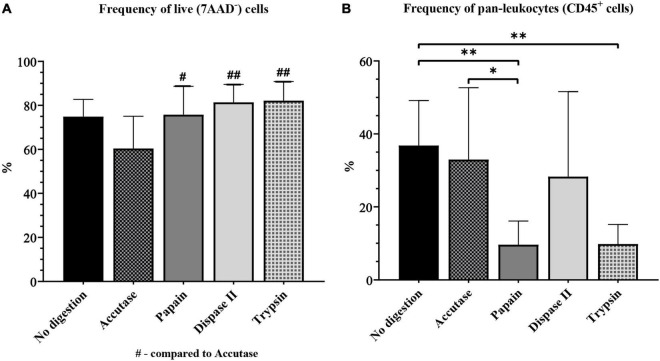
The yield of live cells **(A)** and pan-leukocytes **(B)** on flow cytometry for different enzymatic digestion protocols. */#*P* < 0.05; ^**^/##*P* < 0.01. *N* (mice per group) = 8.

First, we compared the frequency of live cells yielded when different digestion enzymes were used. The frequency of live cells was significantly lower in the accutase group (60.40% ± 4.88) than in the papain (75.77% ± 4.29, *P* = 0.04), dispase II (81.39% ± 2.71, *P* < 0.01), trypsin (82.14% ± 3.28, *P* < 0.01), and control group (74.91 ± 2.77, *P* = 0.07) (Figure 7A).

The frequency of CD45^+^ cells was also significantly higher in the accutase group (32.94% ± 6.58, *P* < 0.05) and the control (36.81% ± 4.37, *P* < 0.01), than in papain (7.62% ± 0.98), and also control when compared to the trypsin group (9.80% ± 2.02, *P* < 0.01). The frequency of CD45^+^ was higher in the dispase II group (28.28% ± 7.77) than in the papain and trypsin group, but was still somewhat lower compared with the accutase and control group (Figure 7B). All of the experimental groups had a high CV (> 25%).

**FIGURE 8:**
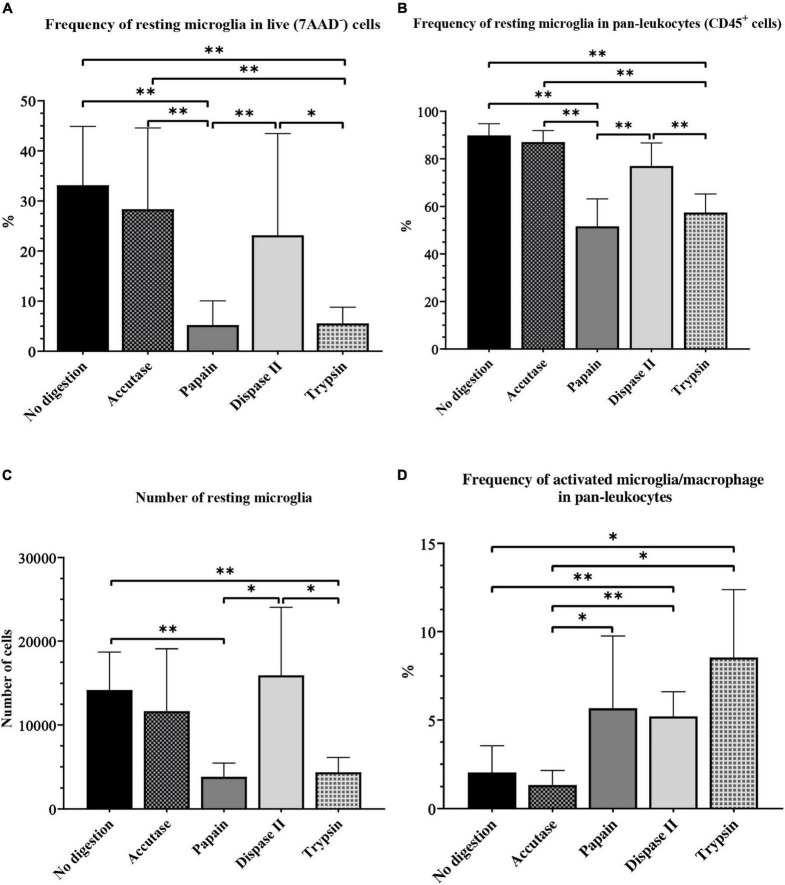
The effect of different enzymatic brain tissue digestion on microglial yield on flow cytometry: The percentage of microglia in the live-cell population **(A)** and pan-leukocytes population **(B)**, absolute number of resting microglia **(C)** and the percentage of activated microglia and macrophages **(D)**. *P < 0.05; ^**^P < 0.01. *N* (mice per group) = 8.

When we assessed the frequency of microglia in live cells and in CD45^+^ cells, the same pattern was observed. The microglial frequency was significantly lower in live cells and in pan-leukocytes population in the papain and trypsin group, compared with the accutase (*P* < 0.01), dispase II (*P* < 0.01) and control group (*P* < 0.01) (Figures 8A,B). The number of microglia in live cells and in pan-leukocytes population was highest in the accutase and control group. In addition, accutase (57.10%) and the control protocol (35.40%) resulted in a smaller CV compared with dispase II protcocol (87.74%) for the percentage of microglia in live cells, which makes them more reliable options. Accutase digestion resulted in the smallest CV for the percentage of microglia in pan-leukocytes (2.61%), followed by dispase II (6.12%) and control protocol (5.47%).

The absolute number of microglia was lowest in the papain (3820 ± 542.9) and trypsin group (4345 ± 667.6) (Figure 8C). However, it was higher in the dispase II group (15948 ± 2697) than in the accutase (11673 ± 2478) and control group (14186 ± 1602), but the differences were not significant. All experimental groups had a high CV (> 25%).

Contrary to our previous results, the frequency of activated microglia and macrophages was highest in the trypsin group (8.55% ± 1.44), followed by the papain (4.46% ± 0.69), and dispase II group (5.21% ± 0.47). This frequency was significantly lower in the accutase (1.08% ± 0.09) and control group (2.03% ± 0.53) compared with other experimental groups (*P* < 0.05) (Figure 8D). However, the accutase protocol had a CV smaller than 25% (23.44%), which makes it the most reliable option.

To sum up, several enzymatic digestion protocols had similar microglial yields, which makes this step far less crucial compared with the myelin removal method. Protocols with accutase, dispase II and protocol where enzymatic digestion was omitted all yielded a similar frequency of microglia. Although the accutase protocol did not result in the highest microglial yield, it was the best digestion enzyme for microglia isolation from the adult mouse brain as it was the least variable method in terms of CV. This translates to lower number of animals needed, which is crucial in animal research. The advantage of using dispase II was the increased frequency of activated microglia and macrophages along with a high yield of resting microglia. The protocol without enzymatic digestion was inexpensive and less time-consuming, yet it still yielded satisfactory results. In our study, the protocol without digestion proved efficient for microglia isolation from healthy mouse brains, however this protocol may not be adequate for brain samples undergoing inflammation. Digestion with papain and trypsin yielded a significantly smaller number of microglia compared with other digestion enzymes. Although we would not recommend the use of these enzymes for resting microglia isolation, they proved efficient for the isolation of activated microglia and macrophages.

#### Characterization of CD11b^+^CD45^int^ cell population

**FIGURE 9:**
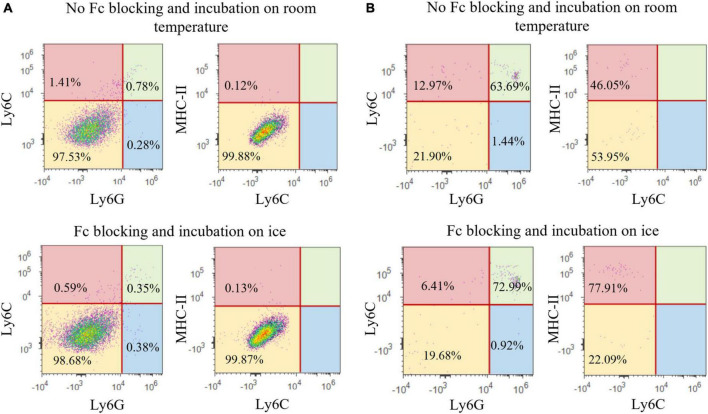
Representative images of Ly6G, Ly6C, and MHC-II expression in CD11b^+^CD45^int^ population **(A)** and CD11b^+^CD45^high^ population **(B)**.

To further characterize the specificity of our protocol for the isolation of microglial cells, we assessed the expression of surface antigens specific for neutrophils, monocytes and dendritic cells (Ly6G, Ly6C, and MHC-II, respectively). In the population of resting microglia (CD11b^+^CD45^int^) 97.53% of the cells did not express Ly6G nor Ly6C. Furthermore, only 0.12% of Ly6G^–^Ly6C^–^ microglia was positive for the MHC-II. Frequency of Ly6G and Ly6C positive cells in the CD11b^+^CD45^int^ population was 1.06 and 2.19%, respectively, while the amount of Ly6G^+^Ly6C^+^ cells was 0.78%. Taken together, these results proved there was no substantial contamination of resting microglia population CD11b^+^CD45^int^ with other immune cells (Figure 9A). This result also confirmed the usage of CD11b and CD45 antibodies as sufficient for defining the population of resting microglia cells in physiological conditions used in our experiments. The lack of other immune cells in the CD11b^+^CD45^int^ cell population was a consequence of using only healthy animals in our research. If the samples that are being processed have some neuroinflammation, the research may benefit from implementing Ly6G, Ly6C, and MHC-II antibodies for identifying resting microglia. On the other hand, the CD11b^+^CD45^high^ cell population had increased expression of Ly6G (65.13%), Ly6C (76.66%), and MHC-II (46.05% of Ly6G^–^Ly6C^–^ cell population), which confirmed it consisted of microglia (21.90%) along with the infiltrated immune cells (Figure 9B).

Furthermore, we assessed whether FBS in the staining media was sufficient for preventing unspecific antibody binding or if the application of Fc blocking solution prior to antibody staining is crucial. Implementing Fc blocking pretreatment did not significantly alter the results, showing that FBS was adequate for preventing non-specific antibody binding (Figure 9). Once again, this finding was probably due to the use of healthy animals in our experiments, and additional blocking of Fc-receptors may need to be implemented for the research of inflammatory processes.

Finally, we assessed the effect of incubation temperature on the microglial yield. In our experiments, incubation was performed at room temperature since the commercial antibodies used already contained a small amount of sodium azide (0.09%), which minimizes internalization and various other adverse effects regardless of incubation temperature (Diamond and DeMaggio, 2000). Additionally, the incubation time was quite short, thus no significant decrease in cell viability was expected. Comparing cell yield of samples incubated at room temperature with the samples incubated on ice showed that, indeed, there were no differences in cell viability nor in the frequency of isolated microglial cells (∼60%). In addition, specificity of the antibody binding was not altered by the incubation temperature and both protocols resulted in the similar yield of Ly6G^+^, Ly6C^+^, and MHC-II^+^ cells (Figure 9).

## Discussion

In the systematic review part of this paper, the analyzed studies showed a consistency in flow cytometry gating strategies and definitions of microglial cell population. All publications reported the same surface markers for microglia identification and separation. The use of animal models was also consistent. Most of the experiments were performed on mice with C57BL/6 genetic background. Although the majority of experiments were carried out only on male mice, quite a number of studies also included female mice (Yang et al., 2017; Cai et al., 2018; Sousa et al., 2018; Kaiser and Feng, 2019; Nugent et al., 2020; Srivastava et al., 2020; Schroeter et al., 2021). Reporting of animal age and sex was not satisfactory, four studies failing to report animal sex (Martin et al., 2017; Calvo et al., 2020; McKinsey et al., 2020; Wang et al., 2020) and six studies failing to report the exact age of animals, although all these studies implied that the experiments were done on adult animals (Martin et al., 2017; Verma et al., 2017; Kaiser and Feng, 2019; McKinsey et al., 2020; Pan and Wan, 2020; Wang et al., 2020). In our experiments, we selected the most commonly used mouse model for flow cytometry: male mice younger than 16 weeks with C57BL/6 genetic background. However, it would be interesting to compare our results with experiments involving animals of different age or sex.

Our experimental data showed that the choice of myelin removal method affected the microglial yield far more than the enzymatic digestion step. The Percoll protocol was the most commonly used myelin removal method in the analyzed publications (Martin et al., 2017; Xu et al., 2017; Yang et al., 2017; Al Mamun et al., 2018, 2020; Cai et al., 2018; Jones et al., 2018; Gao et al., 2019; Kaiser and Feng, 2019; Molina Estevez et al., 2019; Zhou et al., 2019; Brioschi et al., 2020; Calvo et al., 2020; Honarpisheh et al., 2020; McKinsey et al., 2020; Pan and Wan, 2020; Shi et al., 2020; Srivastava et al., 2020; Manrique-Castano et al., 2021; Marino Lee et al., 2021; Piepke et al., 2021; Shan et al., 2021); our experiments confirmed that it was by far the best myelin removal method with the highest microglial yield and optimal CV. Although some studies proved usage of single Percoll gradient is efficient for myelin removal (Jones et al., 2018; Kaiser and Feng, 2019; Molina Estevez et al., 2019; Brioschi et al., 2020; Pan and Wan, 2020; Manrique-Castano et al., 2021), most of the publications used at least 2 different concentration gradients of Percoll for more efficient myelin removal. Therefore, we also implemented this approach in our experimental study by using three Percoll gradients. Overall, the reviewed studies did not use many myelin removal methods–in 32 publications we found only three different methods.

However, the Percoll protocol had the disadvantage of a very high cost compared with the more affordable sucrose and no-removal methods. In addition, it is lengthy and requires additional steps of excess Percoll removal, which results in lower live-cell yield. Both sucrose and no-removal method are more financially accessible and less time consuming, but they are not as efficient in myelin removal and immune cells isolation as the Percoll protocol. Our own protocol for myelin removal using sucrose yielded better results in most of the analyzed categories compared with the no-removal protocol, which makes it a potential low-cost alternative to Percoll.

The literature search identified many different protocols of brain tissue digestion. In most of the studies, a cocktail of more than one digestive enzyme was used. We decided to compare some of the enzymes identified in the literature, but we only used one enzyme per experimental group to eliminate the additive effect of combining different enzymes. The differences in the results of this part of the study were not as clear-cut as those related to the myelin removal protocol. The accutase, dispase II and no-digestion protocols obtained similar microglial yields. The choice between these three methods depends on the purpose and priorities of the research. Accutase was the best option since it had the smallest CV, which makes it the most reliable method. In addition, a smaller CV indicates that fewer animals are needed for obtaining biologically significant results, which is in accordance with ethical guidelines for animals in scientific research (Flecknell, 2002). Although accutase proved to be the most reliable option, the literature search did not identify any publications using accutase for brain tissue digestion. In addition, in the analyzed studies, dispase II was always combined with collagenase (Al Mamun et al., 2018, 2020; Ritzel et al., 2019; Honarpisheh et al., 2020; Kim et al., 2021; Marino Lee et al., 2021).

Regarding enzymatic brain tissue digestion, the qualitative synthesis showed that the most commonly used protocol excluded digestion. Our experimental data corroborated this finding since the no digestion protocol had one of the highest microglial yields. Many analyzed studies failed to report the duration of enzymatic digestion, which makes their experimental protocol harder to repeat. Nevertheless, all studies that performed enzymatic digestion disclosed the concentration of the selected enzymes.

The effectiveness of microglia isolation varied greatly for tissue digestion with different enzymes. In our experiments, tissue digestion with trypsin and papain resulted in the smallest immune cell and microglial yield. Literature suggests that cell digestion with trypsin increases the expression of CD45 surface antigen (Donnenberg et al., 2018), which is also observed in our results. Samples digested with trypsin had, along with papain, the highest frequency of CD11b + CD45^high^ cell population. Additional research is needed to elucidate if papain changes the expression of CD45 surface antigen. Dispase does not affect the expression of CD45 nor CD11b (Autengruber et al., 2012).

We showed how different isolation protocols affect the microglial yield on flow cytometry, and that each protocol had its own advantages and disadvantages. We found that the combination of a myelin removal method and enzymatic tissue digestion works best for microglia isolation from healthy adult mice. The proposed practical protocol based on the study results comprises of Percoll as the best myelin removal method, and the use of accutase for the enzymatic digestion step. Furthermore, we efficiently identified resting microglia by simple panel including only CD45 and CD11b markers, and confirmed that CD11b^+^CD45^int^ population does not contain contaminating immune cells (Ly6G^+^, Ly6C^+^) or activated microglia (MHC II^+^) (Wolf et al., 2018; Gopinath et al., 2020). Eventual further research would be needed to determine if our protocol is the best option for microglia isolation from diseased brain tissue (for example brain stroke, encephalitis, etc.) or from the perinatal brain.

## Data availability statement

The original contributions presented in this study are included in the article/Supplementary material, further inquiries can be directed to the corresponding author.

## Ethics statement

The animal study was reviewed and approved by School of Medicine, University of Zagreb.

## Author contributions

SS, PJ, SG, DG, and AG contributed to the conception and design of the study. ST organized the database and performed the statistical analysis. SS wrote the first draft of the manuscript. PJ, ST, and AG wrote sections of the manuscript. All authors contributed to the manuscript revision, read, and approved the submitted version.
